# HPV16 E6 Promotes Breast Cancer Proliferation via Upregulation of COX-2 Expression

**DOI:** 10.1155/2017/2948467

**Published:** 2017-11-09

**Authors:** Y. X. Wang, Y. Z. Li, Z. Y. Zhang, J. Q. Wang, J. Cui, X. L. Qian

**Affiliations:** ^1^Department of Pathology, School of Basic Medical Sciences, Xinxiang Medical University, Xinxiang 453003, China; ^2^The Third Affiliated Hospital of Xinxiang Medical University, Xinxiang 453003, China

## Abstract

*Background. *Breast cancer remains the leading cause of cancer-related mortality worldwide. It has been indicated that human papillomaviruses 16 (HPV16) might participate in the pathogenesis and development of breast cancer. However, the detected rate of HPV16 varies with region. We will investigate HPV16 E6 expression in North China and explore the effects and mechanism of HPV16 E6 on breast cancer proliferation in this study.* Methods.* The expressions of HPV16 E6 and COX-2 in paraffin-embedded tissues of the invasive ductal breast cancer were detected by qPCR and IHC. The effects of HPV16 E6 on breast cancer proliferation were determined by function studies. The mechanism of HPV16 E6 in promoting breast cancer proliferation was explored by Western blot and Dual-Luciferase Reporter Assay.* Results. *HPV16 E6 was positive in 28% invasive ductal breast carcinoma in North China; HPV16 E6 promoted breast cancer proliferation. Inhibition of COX-2 by siCOX-2 or Celecoxib attenuated the proliferation of breast cancer cells with HPV16 E6 expression; and the upregulation of COX-2 could be suppressed by the inhibition of NF-*κ*B activity.* Conclusion.* HPV16 E6 promotes breast cancer proliferation by activation of NF-*κ*B signaling pathway and increase of COX-2 expression. COX-2 will be a potential target for HPV16 E6-associated breast cancer.

## 1. Introduction

Breast cancer claims most deaths of women suffering from cancer worldwide; the incidence of breast cancer and mortality of cases with carcinoma has increased gradually in recent years [1, 2]. It has been proven that the incidence of human breast cancer is related to such factors as lifestyle, medical conditions, oncogenic genes, and virus infection [3–8]. Human papillomaviruses (HPVs) are small DNA viruses with high affinity for epithelia. HPVs are classified into low risk (LR) and high risk (HR) according to their pathogenicity. Among the HPV genotype of high risk, HPV16 is the most prevalent. The integration of HPV16 DNA promotes a constitutive high expression of E6 oncoproteins and results in the extensive proliferation of the infected epithelial cells. Recently, HR-HPVs, especially HPV16, has been indicated to be involved in the pathogenesis and development of breast cancer. However, the detection rate varies with regions [9, 10]. Some prophylactic vaccines to prevent the cancer by HPV16 have been developed [11]. Unfortunately, their effects are still unsatisfactory to most of the cases infected with HPV.

Cyclooxygenase-2 (COX-2) is an important isoform of COX which functions as catalyzer in the synthesis of prostaglandins (PG) from arachidonic acid [12]. COX-2 is undetectable in most normal tissues but is upregulated in many malignancies, including breast cancer [13]. A strong correlation between COX-2 and carcinogenesis has been confirmed by carcinogen-induced animal models in recent studies, which indicates that COX-2 plays an important role in tumor promotion. COX-2 can promote the proliferation, angiogenesis, invasiveness, metastasis and inhibition of apoptosis, and immunosurveillance [14]. Celecoxib is a selective COX-2 inhibitor, and it can also be used to treat cancers of some kinds [15–17]. Although COX-2 is overexpressed in HPV-induced lesions, it is still uncertain whether the upregulation of COX-2 results from HPV16 E6 in breast cancer.

In this study, the expression and relationship of HPV16 E6 and COX2 DNA in paraffin-embedded invasive ductal breast cancer in the north of China will be explored and whether COX-2 is the target of HPV16 E6 or not will be made clear. Therefore, this study is important to the improvement of carcinogenic mechanism of HPV16 E6 and exploration of new therapeutic target for HPV16 E6-associated cancers.

## 2. Materials and Methods

### 2.1. Tissue Specimens

50 cases of invasive ductal breast cancer samples were collected from Department of Pathology, the Third Affiliated Hospital of Xinxiang Medical University (Xinxiang, China), from January 2010 to December 2011. All the cases were female and had no chemotherapy, radiotherapy, and immunotherapy history. The patients are in the 25–85 age bracket (55.5 ± 14.4). All the samples were fixed in buffered formalin, embedded in paraffin, and stored at room temperature for later use. And the samples had been diagnosed with invasive ductal breast cancer by two pathologists on the basis of Hematoxylin-Eosin (HE) staining sections. This study was approved by the ethics committee of Xinxiang Medical University Institutional Board (Xinxiang, China).

### 2.2. DNA Isolation and qPCR

10 pieces of 4 *μ*m paraffin sections were collected from each sample for DNA isolation. During cutting, the first 3 sections and the excess paraffin were discarded, and the remainder was gathered in a 1.5 mL tube. DNA was extracted with QIAamp DNA FFPE Tissue Kit (QIAGEN, Germany) from the paraffin-embedded tissues according to the manufacturer's instruction. Firstly, the sections were disposed of with xylene for deparaffinization (3 × 10 min) and then were rehydrated through graded ethanol steps. Next, the samples were dried at room temperature and later incubated overnight at 56°C with Proteinase K. On the next day, the samples were incubated for 1 hour at 90°C and then the mixture of AL Buffer and 100% ethanol was added. After brief spinning, the entire lysate was transferred to the QIAamp MinElute column, washed twice, and centrifuged (1400 rpm, 3 min). Lastly, DNA was eluted from the column by ATE buffer. HPV16 E6 and COX-2 DNA were detected by qPCR by the Applied Biosystems 7500 Sequence Detection system, using SYBR Green I. The CT values above 35 were not acceptable. And the valid data were normalized to the geometric mean of housekeeping gene GAPDH and calculated as 2^–ΔΔCT^. The primers for qPCR were shown in Table 1. The cycling conditions were as follows: initial denaturation for 1 min at 95°C, 45 cycles of denaturation at 95°C for 30 sec, annealing at 50°C for 45 sec, extension at 72°C for 30 sec, and the last extension at 72°C for 5 min.

### 2.3. Immunohistochemistry

The expressions of HPV16 E6 and COX-2 protein in breast invasive ductal carcinoma samples were detected by SP immunohistochemistry (SP-9000, ZSGB-BIO, China). Paraffin-embedded specimens were firstly cut into 4 um sections and baked at 60°C for 1 hour, deparaffinized with xylenes, and rehydrated with graded ethanol. After incubation in 3% H_2_O_2_ to quench the endogenous peroxidase activity, the sections were heated in 0.01 M, pH 6.0, sodium citrate buffer for antigenic retrieval. Later, the sections were blocked with normal nonimmune serum for 20 min and then incubated with rabbit anti-HPV16 E6 (1 : 50; Santa Cruz, USA) or rabbit anti-COX-2 (1 : 100; Proteintech, USA) overnight at 4°C. At the next day, the sections were treated with universal type of secondary antibody and followed with streptavidin-horseradish peroxidase complex. Diaminobenzidine (DAB, ZSGB-BIO, China) was used for color development. PBS was used to replace primary antibody as negative contrast. Finally, the stained slides were evaluated independently by two pathologists who were blind to the clinical parameters. The positive tumor cells were stained in the nucleus for HPV16 E6 protein. The IHC staining of COX-2 was done in the cytoplasm and assessed as high expression or low expression according to the IRS scores [18].

### 2.4. Cell Culture and Western Blot

The stable cells of MCF-7/HPV16 E6 and MCF-7/Vector established in our previous study were cultured in DMEM (Invitrogen). The medium was supplemented with 10% fetal bovine serum (FBS, Gibco) and 1% penicillin/streptomycin (Invitrogen). Protein lysates obtained from the cells were resolved on 10.5% SDS polyacrylamide gel and electrotransferred to polyvinylidene difluoride (PVDF, Merck Millipore) membranes. Later, the PVDF membranes were treated with 5% nonfat dry milk and then incubated with rabbit anti-HPV16 E6 (1 : 200; Santa Cruz, USA), rabbit anti-COX-2 (1 : 200; proteintech, USA), or mouse a-tubulin (1 : 2000; Cell Signaling Technology, USA) overnight at 4°C. At last, the membranes were incubated with the appropriate secondary antibodies HRP-conjugated anti-rabbit IgG (1 : 5000, CST, USA) or HRP-conjugated anti-mouse IgG (1 : 5000, CST, USA) and detected by the chemiluminescence imaging analysis system (Tanon, China).

### 2.5. MTT Assay, Colony Formation Assay, and Soft Agar Assay

#### 2.5.1. MTT Assay

1 × 10^3^ cells were seeded on 96-well plates and cultured for 24 h. 20 *μ*l 5 g/l 3-(4,5-dimethylthiazol-z-yl)-2,5-diphenyltetrazolium bromide (MTT, Sigma, USA) was added to each well and incubated for 4 h. Then, MTT was removed and 150 *μ*l dimethylsulfoxide (DMSO; Sigma, USA) was added to the wells. The absorbance was measured at 450 nm with a microplate autoreader (Bio-Rad, Hercules, CA, USA). The experiment was conducted repeatedly for three times.

#### 2.5.2. Colony Formation Assay

Cells were trypsinized and plated on 6-well plates (200 cells/well) and cultured for 2 weeks. The colonies were stained with Hematoxylin for 30 min after fixation with 4% paraformaldehyde for 5 minutes. The number of colonies, defined as >50 cells/colony, was counted. Three independent experiments were performed.

#### 2.5.3. Soft Agar Assay

Six-well plates were covered with a layer of 0.6% agar (Sigma, USA) in medium supplemented with 20% fetal bovine serum. Cells were prepared in 0.3% agar and seeded in triplicate at a dilution of 1 × 10^3^. The plates were incubated at 37°C in a humid atmosphere of 5% CO_2_ for 2 weeks. Each experiment was repeated at least 3 times. Colonies were photographed after 2 weeks at an original magnification of ×200.

### 2.6. Tumorigenesis in Nude Mice

4–6-week-old BABL/c nude mice were purchased from the Center of Laboratory Animal Science of Guangdong (Guangzhou, China). All animal experiments were conducted in accordance with current Chinese regulations and standards regarding the use of laboratory animals, and all animal procedures were approved by the Xinxiang Medical University Institutional Animal Care and Use Committee. Xenograft tumors were generated by subcutaneous injection of 2 × 10^6^ stable cells of MCF7/HPV16 E6 and MCF7/Vector (*n* = 6) on the hindlimbs. Tumor size was measured by a slide caliper twice weekly (volume = length × width × height). All mice were euthanized 3 weeks later and the tumors were fixed and 4 *μ*m sections were cut and stained with Hematoxylin and Eosin according to standard protocols. Sections were further under IHC staining by antibody against Ki-67.

### 2.7. SiRNA, Celecoxib, and PDTC Treatment

COX-2 siRNA (the target sequence: GCTCAGCCATACAGCAAAT, Ribob Biotechnology, China) was transfected into the cells by Lipofectamine 2000 Reagent (Invitrogen). Or the cells were treated with Celecoxib (Selleck Chemicals) at a concentration of 1 mol/L which had been diluted by dimethylsulfoxide (DMSO, Sigma) for 48 h. Then the cells were used for protein collection or functional experiments. The cells were treated by the NF-*κ*B inhibitor of pyrrolidinedithiocarbamate (PDTC, Beyotime Institute of Biotechnology) at a concentration of 0.5 mol/L which had been diluted by DMSO for 48 h and then the cells were used for the following experiments.

### 2.8. Dual-Luciferase Reporter Assay

Cells at 60% confluence in 24-well plate were cotransfected with the NF-*κ*B Luciferase Reporter (Beyotime Institute of Biotechnology, China), the Renilla Luciferase Reporter Vector pRL-TK (Promega), and the Vector or HPV16 E6 plasmids by lipofectamine 2000. 48 hours later, the Passive Lysis Buffer (PLB) was added to lyse and then was collected. Luciferase Assay Reagent II (LAR II) was added to measure the firefly luminescent signal. Then the reaction was quenched, and the Renilla Luminescence was detected immediately after adding Stop & Glo Reagent. All of the experiments were conducted three times.

### 2.9. Statistical Analysis

All statistical analyses were performed by SPSS 20.0 for Windows. The data were expressed as means ± standard deviations from at least three independent experiments. Student's* t*-test was conducted for the analysis of the two groups. The Spearman correlation was used to analyze the correlation between HPV16 E6 and COX-2 expression. *p* < 0.05 was considered significant. Statistically significant data were indicated by asterisks: ^*∗*^(*p* < 0.05), ^*∗∗*^(*p* < 0.01).

## 3. Results 

### 3.1. Expression of HPV16 E6 DNA in Invasive Ductal Breast Cancer

The results of HPV16 E6 detection by qPCR in 50 cases of paraffin-embedded invasive ductal breast cancer samples showed that it was positive in 14 samples (28%) (Table 2).

### 3.2. HPV16 E6 Promotes the Proliferation of Breast Cancer Cell In Vitro and In Vivo

**Figure 1:**
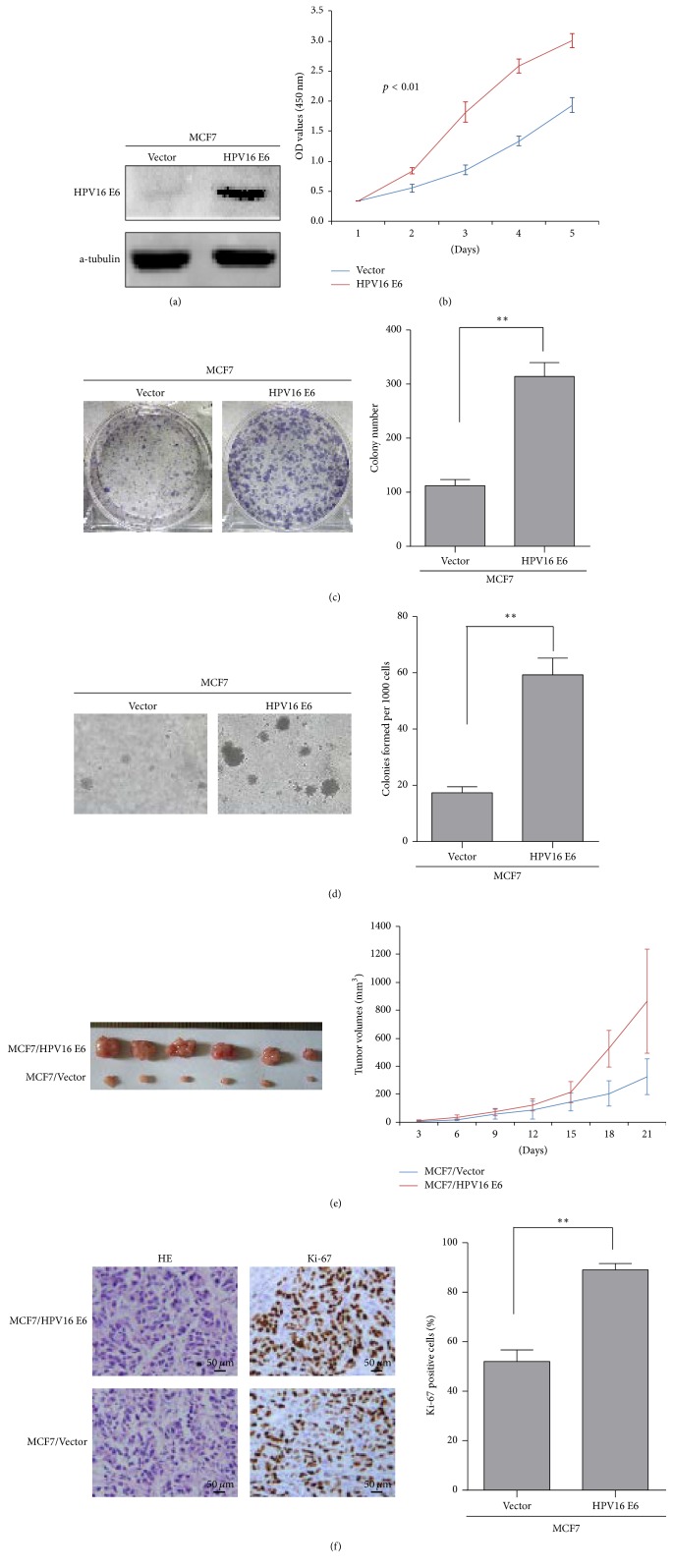
*HPV16 E6 promotes the proliferation of breast cancer.* (a) The expression of HPV16 E6 in MCF-7/HPV16 E6 and MCF-7/Vector by Western blot. (b)–(d) The proliferative ability of the indicated cells detected by MTT assays, colony formation assays, and soft agar assays. Only cell colonies containing more than 50 cells were counted. Error bars represent mean ± SD from 3 independent experiments. ^*∗∗*^*p* < 0.01. (e) MCF-7/HPV16 E6 and MCF-7/Vector cells were injected into the hind limbs of nude mice (*n* = 6). Tumor volumes were measured on the indicated days. The tumor volume data were presented as the mean ± SD. (f) Histopathological analyses of xenograft tumor. The tumor sections were stained with H&E or subjected to IHC staining using an antibody against Ki-67. Error bars represent mean ± SD from three independent experiments. ^*∗∗*^*p* < 0.01.

We firstly examined the expression of HPV16 E6 protein in the stable cells of MCF-7/HPV16 E6 and MCF-7/Vector established in our previous study (Figure 1(a)). MTT assays revealed that the expression of HPV16 E6 significantly increased the growth of MCF-7 cells compared with the vector-transfected cells (Figure 1(b)). The results of colony formation assays and soft agar assays showed that the expression of HPV16 E6 significantly promoted the proliferation of MCF-7 cells in vitro (Figures 1(c) and 1(d)). The tumorigenesis assays in nude mice demonstrated that the tumors in the MCF7/HPV16 E6 group grew much faster than those in the MCF7/Vector group (Figure 1(e)). Furthermore, IHC confirmed that the tumors of the MCF7/HPV16 E6 group showed much higher Ki-67 index than that in MCF7/Vector group (Figure 1(f)).

### 3.3. HPV16 E6 Promotes the Proliferation of Breast Cancer by Upregulating COX-2 Expression

**Figure 2:**
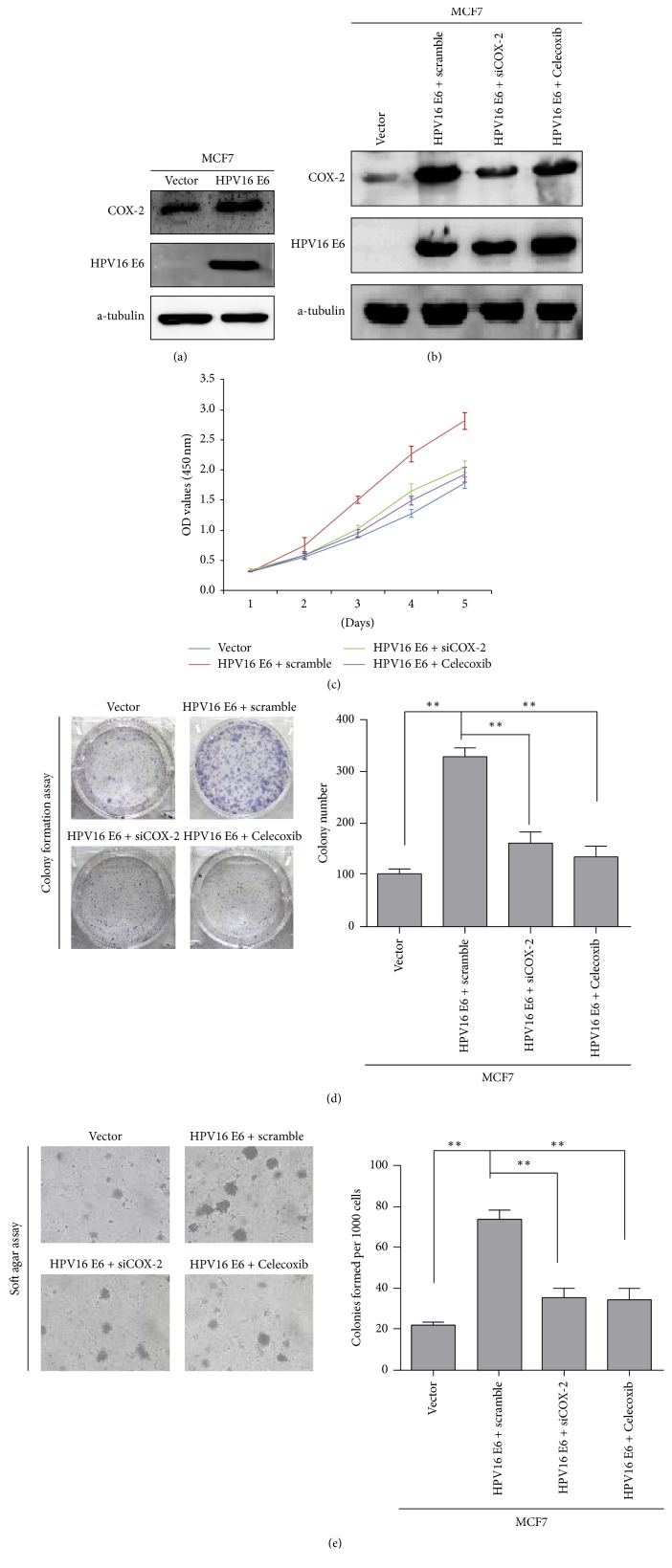
*HPV16 E6 promotes the proliferation of breast cancer by upregulating COX-2 expression. *(a) The expression of HPV16 E6 and COX-2 expression in MCF-7/HPV16 E6 and MCF-7/Vector cells. (b) The construction of cells with HPV16 E6 expression and COX-2 suppression which induced by siCOX-2 and Celecoxib. (c)–(e) The proliferative ability of the indicated cells detected by MTT assays, colony formation assays, and soft agar assays. Only cell colonies containing more than 50 cells were counted. Error bars represent mean ± SD from 3 independent experiments. ^*∗∗*^*p* < 0.01.

We examined the protein expression of COX-2 in MCF-7/HPV16 E6 and MCF-7/Vector cells and found that the expression of COX-2 increased with the expression of HPV16 E6 (Figure 2(a)). Then, we treated MCF-7/HPV16 E6 cells with siCOX-2 and Celecoxib to suppress the expression of COX-2. A series of functional experiments were performed with these cells (Figure 2(b)). The results of MTT, colony formation, and soft agar assays showed that the suppression of COX-2 significantly inhibited the proliferation of MCF-7/HPV16 E6 cells (Figures 2(c)–2(e)). All of these results demonstrated that HPV16 E6 promoted breast cancer proliferation by upregulation COX-2 expression.

### 3.4. HPV16 E6 Expression Positively Correlates with COX-2 Expression in Breast Cancer

**Figure 3:**
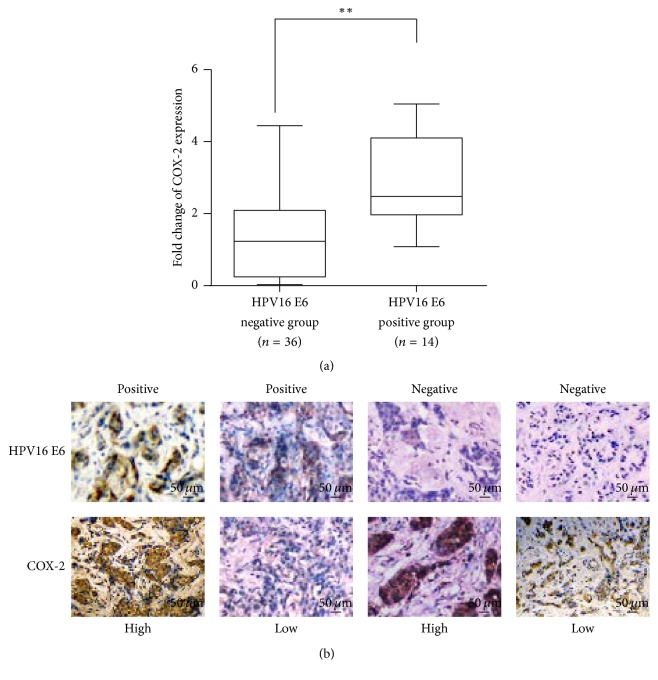
*HPV16 E6 expression positively correlates with COX-2 expression in breast cancer. *(a) The expression of COX-2 DNA in HPV16 E6 positive and negative invasive ductal breast cancer samples by qPCR analysis (2^–ΔΔCT^). ^*∗∗*^*p* < 0.01. (b) The representative images of HPV16 E6 and COX-2 expression in invasive ductal breast carcinoma tissues by IHC.

We detected the expression of COX-2 DNA in the same 50 cases of paraffin-embedded invasive ductal breast cancer samples by qPCR and found that the expression level of COX-2 in HPV16 E6 positive samples was significantly higher than that in HPV16 E6 negative samples (Figure 3(a)) (Table 2). In addition, HPV16 E6 and COX-2 protein were detected in these 50 cases of paraffin-embedded breast invasive ductal carcinoma samples by IHC (Figure 3(b)). The HPV16 E6 protein was expressed in 14 cases, which was in accordance with the qPCR results. The Spearman correlation analysis showed that there was positive correlation between HPV16 E6 and COX-2 expression in breast invasive ductal carcinoma (Table 3).

### 3.5. HPV16 E6 Upregulates COX2 Expression by Activating NF-*κ*B Signaling Pathway

**Figure 4:**
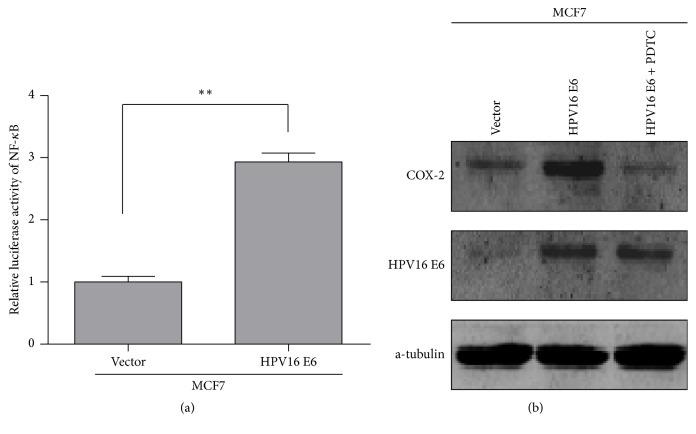
*HPV16 E6 upregulates COX2 expression by activating NF-κB signaling pathway.* (a) The luciferase activity of NF-*κ*B in MCF-7 cells with the expression of HPV16 E6. ^*∗∗*^*p* < 0.01. (b) The expression of COX-2 protein in MCF-7 cells with different treatment.

The activity of NF-*κ*B signaling pathway was found increased significantly by the expression of HPV16 E6 in MCF-7/HPV16 E6 than that in MCF-7/Vector cells (Figure 4(a)). And the overexpression of COX-2 induced by the expression of HPV16 E6 in MCF-7/HPV16 E6 cells could be suppressed after being treated with PDTC (Figure 4(b)).

## 4. Discussion

It has been reported that HPV could induce a range of benign and malignant lesions, especially high-risk HPV types such as HPV16 and HPV18, which are closely correlated with the occurrence of some malignant lesions. For example, both epidemiologic and experimental evidence had demonstrated that some HR-HPVs could induce cervical cancer. HPV16 could encode E6 oncoprotein, which had effects on the carcinogenesis, proliferation, invasion, and metastasis. However, the association of HPV16 with breast cancer varies widely. It was first reported that HPV16 could immortalize normal human mammary epithelial cells and reduce their growth factor requirements [19]. Up to now, HPV16 has been repeatedly detected in 0 to 86% of breast cancer which indicated an inconsistent association of HPV16 with breast cancer. But the recent meta-analysis does suggest an increased risk of breast cancer by HPV infection [20].

In this study, we detected the HPV16 E6 DNA in breast invasive ductal carcinoma samples and found that it could be detected in 28% of them. That is to say, HPV16 E6 might be an important factor to breast cancer in the north of China. Also we found that the infection of HPV16 E6 could increase the proliferation of breast cancer. It is important to explore molecular targets for gene therapy of HPV16 E6-associated breast cancer. Although there had been some efficacious vaccines for the prevention of HPV infection, they do not work for those already infected with HPV. Therefore, further studies are necessary to develop better therapeutic options for the treatment of HPV infection. In our study, we found that the expression of COX-2 in HPV16 E6 positive breast invasive ductal carcinoma cases was much higher than that in HPV16 E6 negative ones. This result indicated that the expression of COX-2 could be upregulated by the expression of HPV16 E6, which agrees with the previous studies in other cancers [21, 22]. COX-2 is the key rate-limiting enzyme in PGE2 synthesis and plays critical roles in tumor associated immune dysfunction and cancer progression [23, 24]. We inferred that COX-2 might be an important target for HPV16 E6-associated breast cancer.

In the following study, we found that the suppression of COX-2 significantly inhibited the proliferation of MCF-7/HPV16 E6 cells. The results demonstrated that the effects of HPV16 E6 in promoting breast cancer proliferation were achieved by the upregulation of COX-2. It has been reported that COX-2 plays important roles in the carcinogenesis and development of many tumors including breast cancer [25–27]. In the past few years, The animal experiments in vivo clearly indicate that high COX-2 expression is correlated to the genesis of mammary tumors that are sensitive to treatment with nonselective and selective COX-2 inhibitors. Recently, the combination of specific COX-2 inhibitors with conventional chemotherapy as a novel approach brings about some promising changes in the field of breast cancer treatment. Celecoxib is a selective COX2 inhibitor which has been used in the therapy of osteoarthritis and rheumatoid arthritis. And now, it has been proven that Celecoxib could prevent carcinogenesis, delay cancer progression, and enhance the efficacy of conventional cancer therapies, such as chemotherapy and radiation therapy [28–31]. For example, nimotuzumab and Celecoxib exert synergistic antiproliferation effects in breast cancer [32]; adjuvant use of Celecoxib and lanreotide is beneficial for advanced hepatocellular carcinoma [33]. Therefore, more and more COX-2 inhibitors with improved anticancer activity are being developed [34–36]. Currently, there were no effective therapeutic methods for established HPV cancers [37]. Thus, it is urgent to develop effective therapeutic methods for treating HPV-induced cancers. It has been shown that Celecoxib-treated HPV16 +/− animals show reduced incidence of epidermal dysplasia than untreated mice [38]. Celecoxib was also reported to significantly decrease breast tumor volume in rats [39]. In conclusion, the use of Celecoxib might be an effective therapeutic method for HPV-associated breast cancer.

NF-*κ*B, a pivotal transcriptional factor in cancer cells, participates in the tumorigenesis and progression by increasing proliferation, regulating angiogenesis and suppressing apoptosis. It has been reported that some E6/E7-regulated gene products are target genes of NF-*κ*B, a dimeric transcription factor involved in the expression of proteins necessary for innate immunity, apoptosis, and cell proliferation. And accumulating studies have demonstrated that NF-*κ*B plays an important role in the regulation of COX-2 expression [40–42]. Therefore, we postulated whether the upregulation of COX-2 medicated by HPV16 E6 was achieved by the activation of NF-*κ*B signaling pathway. Our study finally confirmed that HPV16 E6 can upregulate COX-2 expression by activating NF-*κ*B signaling pathway, which is in accordance with previous result [43].

Overall, our current study showed the presence of HPV16 E6 DNA in collected clinical biopsy sections from breast cancers (28%), and the HPV16 E6 promoted the proliferation of breast cancer via activating NF-*κ*B signaling pathway and upregulated the expression of COX-2. In addition, inhibition of COX-2 by siCOX-2 or Celecoxib attenuated the proliferation of breast cancer cells with HPV16 E6 expression. In conclusion, this study may provide a potent therapeutic target for HPV16 E6-associated breast cancer.

## Figures and Tables

**Table 1:** Primer sequences used for qPCR.

Gene	Forward primer	Reverse primer
HPV16E6	GTATGGAACAACATTAGAACAGCAA	GTGGCTTTTGACAGTTAATACACC
COX-2	CGAGGTGTATGTATGAGTGT	AGTGGGTAAGTATGTAGTGC
GAPDH	GACTCATGACCACAGTCCATGC	AGAGGCAGGGATGATGTTCTG

**Table 2:** HPV16 E6 and COX-2 DNA expression in invasive ductal breast cancer.

Sample	Gender	Age	CT GAPDH (mean ± SD)	CT of HPV16E6 (mean ± SD)	CT of COX-2 (mean ± SD)
1	Female	61	25.15 ± 0.07	Undetermined	27.56 ± 0.43
2	Female	35	24.27 ± 0.18	26.26 ± 0.09	23.62 ± 0.23
3	Female	39	25.51 ± 0.32	Undetermined	27.68 ± 0.39
4	Female	59	23.55 ± 0.18	24.53 ± 0.17	21.38 ± 0.38
5	Female	35	26.42 ± 0.23	26.54 ± 0.29	25.36 ± 0.24
6	Female	73	26.09 ± 0.32	Undetermined	28.29 ± 0.10
7	Female	50	28.07 ± 0.07	Undetermined	27.18 ± 0.11
8	Female	64	24.58 ± 0.20	30.25 ± 0.17	23.22 ± 0.19
9	Female	62	26.94 ± 0.05	Undetermined	29.52 ± 0.18
10	Female	63	27.59 ± 0.50	Undetermined	25.67 ± 0.34
11	Female	63	25.07 ± 0.27	Undetermined	27.77 ± 0.35
12	Female	66	29.35 ± 0.16	Undetermined	28.77 ± 0.44
13	Female	51	25.06 ± 0.12	Undetermined	27.53 ± 0.41
14	Female	43	21.30 ± 0.21	Undetermined	26.64 ± 0.91
15	Female	45	25.44 ± 0.26	Undetermined	27.56 ± 0.29
16	Female	68	24.33 ± 0.15	35.77 ± 0.12	24.21 ± 0.17
17	Female	74	26.49 ± 0.30	Undetermined	28.32 ± 0.21
18	Female	42	26.28 ± 0.19	28.41 ± 0.30	24.42 ± 0.15
19	Female	53	28.28 ± 0.22	Undetermined	27.30 ± 0.10
20	Female	44	27.51 ± 0.40	Undetermined	28.52 ± 0.18
21	Female	53	26.62 ± 0.20	32.35 ± 0.21	26.44 ± 0.20
22	Female	41	27.60 ± 0.50	Undetermined	26.52 ± 0.23
23	Female	59	24.57 ± 0.23	Undetermined	27.58 ± 0.21
24	Female	42	29.44 ± 0.23	Undetermined	29.13 ± 0.18
25	Female	44	25.67 ± 0.17	Undetermined	24.55 ± 0.37
26	Female	33	24.67 ± 0.20	23.48 ± 0.21	22.33 ± 0.17
27	Female	71	27.36 ± 0.15	Undetermined	27.52 ± 0.37
28	Female	60	25.19 ± 0.13	29.36 ± 0.15	24.12 ± 0.03
29	Female	54	29.34 ± 0.27	Undetermined	27.19 ± 0.29
30	Female	32	25.52 ± 0.26	28.45 ± 0.10	23.56 ± 0.28
31	Female	80	24.81 ± 0.15	Undetermined	24.15 ± 0.09
32	Female	53	26.41 ± 0.19	Undetermined	27.45 ± 0.31
33	Female	85	23.28 ± 0.10	Undetermined	21.67 ± 0.10
34	Female	43	28.76 ± 0.30	Undetermined	28.29 ± 0.15
35	Female	60	24.54 ± 0.28	27.62 ± 0.26	22.59 ± 0.13
36	Female	61	29.60 ± 0.36	Undetermined	29.33 ± 0.29
37	Female	77	27.57 ± 0.31	Undetermined	26.47 ± 0.15
38	Female	56	29.82 ± 0.03	Undetermined	29.21 ± 0.04
39	Female	59	24.70 ± 0.18	31.44 ± 0.18	22.49 ± 0.35
40	Female	32	22.80 ± 0.16	28.41 ± 0.15	21.59 ± 0.11
41	Female	68	26.29 ± 0.18	34.51 ± 0.34	25.67 ± 0.10
42	Female	64	27.37 ± 0.48	Undetermined	26.29 ± 0.15
43	Female	60	27.95 ± 0.02	Undetermined	26.59 ± 0.13
44	Female	58	30.49 ± 0.17	Undetermined	30.39 ± 0.23
45	Female	78	26.27 ± 0.42	Undetermined	24.74 ± 0.55
46	Female	60	27.44 ± 0.34	Undetermined	27.24 ± 0.22
47	Female	58	29.74 ± 0.16	Undetermined	29.38 ± 0.24
48	Female	42	22.42 ± 0.25	31.48 ± 0.39	21.32 ± 0.22
49	Female	25	23.71 ± 0.20	Undetermined	23.45 ± 0.22
50	Female	57	23.61 ± 0.44	Undetermined	25.42 ± 0.52

**Table 3:** Correlation between HPV16 E6 and COX-2 expression in invasive ductal breast cancer tissues by IHC.

HPV16 E6 expression	COX-2 expression		*r* value	*p* value
	High	Low		
Positive	12	2	0.327	0.020
Negative	18	18		
